# Overexpression of Three Glucosinolate Biosynthesis Genes in *Brassica napus* Identifies Enhanced Resistance to *Sclerotinia sclerotiorum* and *Botrytis cinerea*

**DOI:** 10.1371/journal.pone.0140491

**Published:** 2015-10-14

**Authors:** Yuanyuan Zhang, Dongxin Huai, Qingyong Yang, Yan Cheng, Ming Ma, Daniel J. Kliebenstein, Yongming Zhou

**Affiliations:** 1 National Key Laboratory of Crop Genetic Improvement, College of Plant Science and Technology, Huazhong Agricultural University, Wuhan 430070, China; 2 Department of Plant Sciences, University of California Davis, One Shield Avenue, Davis, CA 95616, United States of America; 3 DynaMo Center of Excellence, Copenhagen Plant Science Centre, University of Copenhagen, Thorvaldsensvej 40, DK-1871 Frederiksberg C, Denmark; Chungnam National University, REPUBLIC OF KOREA

## Abstract

*Sclerotinia sclerotiorum* and *Botrytis cinerea* are notorious plant pathogenic fungi with an extensive host range including Brassica crops. Glucosinolates (GSLs) are an important group of secondary metabolites characteristic of the Brassicales order, whose degradation products are proving to be increasingly important in plant protection. Enhancing the defense effect of GSL and their associated degradation products is an attractive strategy to strengthen the resistance of plants by transgenic approaches. We generated the lines of *Brassica napus* with three biosynthesis genes involved in GSL metabolic pathway (*BnMAM1*, *BnCYP83A1* and *BnUGT74B1*), respectively. We then measured the foliar GSLs of each transgenic lines and inoculated them with *S*. *sclerotiorum* and *B*. *cinerea*. Compared with the wild type control, over-expressing *BnUGT74B1* in *B*. *napus* increased the aliphatic and indolic GSL levels by 1.7 and 1.5 folds in leaves respectively; while over-expressing *BnMAM1* or *BnCYP83A1* resulted in an approximate 1.5-fold higher only in the aliphatic GSL level in leaves. The results of plant inoculation demonstrated that *BnUGT74B1*-overexpressing lines showed less severe disease symptoms and tissue damage compared with the wild type control, but *BnMAM1* or *BnCYP83A1*-overexpressing lines showed no significant difference in comparison to the controls. These results suggest that the resistance to *S*. *sclerotiorum* and *B*. *cinerea* in *B*. *napus* could be enhanced through tailoring the GSL profiles by transgenic approaches or molecular breeding, which provides useful information to assist plant breeders to design improved breeding strategies.

## Introduction

*Sclerotinia sclerotiorum* and *Botrytis cinerea*, which are closely related necrotrophic plant pathogenic fungi, are notable for their wide host ranges and environmental persistence [1, 2]. Both of them cause the rotting of leaves, stems and pods, resulting in vast economic damages in agricultural crops, especially in Brassica oil crops [3, 4], which produce approximate 72.5 million tonnes of oilseeds worldwide in 2013 (FAOSTAT data 2013, http://faostat.fao.org/site/567/DesktopDefault.aspx?PageID=567#ancor). For example, infection of *B*. *napus* caused by *S*. *sclerotiorum* causes 10%–20% of yield loss every year in China, and the yield loss can be up to 80% in severely infected fields [5]. Chemical control and their application not only cause environmental contamination but also lack the suitable forecasting methods to enable the timely application of fungicides. Therefore, breeding resistance to the two fungi in this crop is an effective approach to reduce crop losses. However, the progress of such an effort is slow due to the complex interactions between necrotrophs and their host plants [6] as well as the lack of resistant germplasm [5].

**Fig 1:**
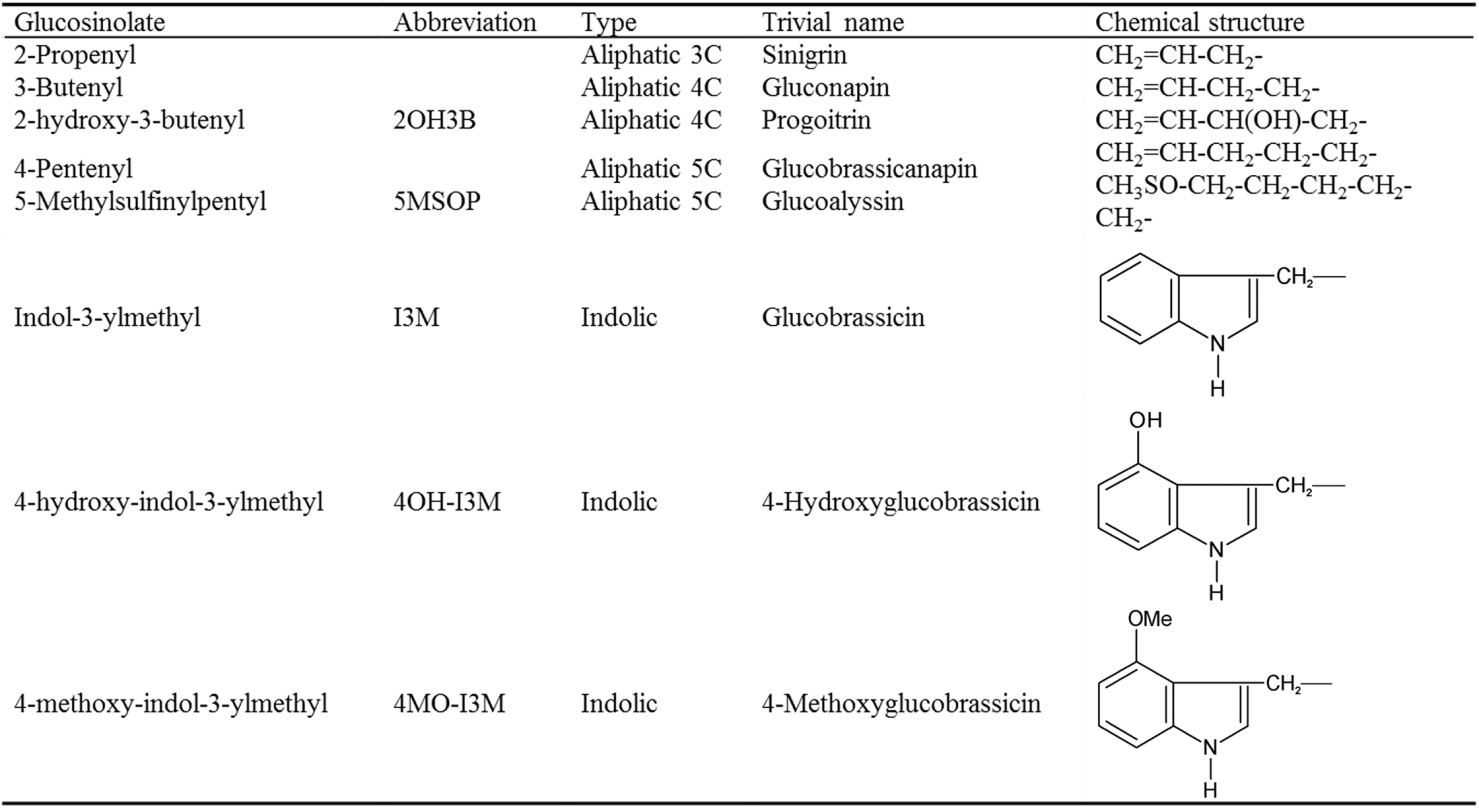
Abbreviations, trivial names and side-chain structures of the glucosinolates detected in this study.

**Fig 2:**
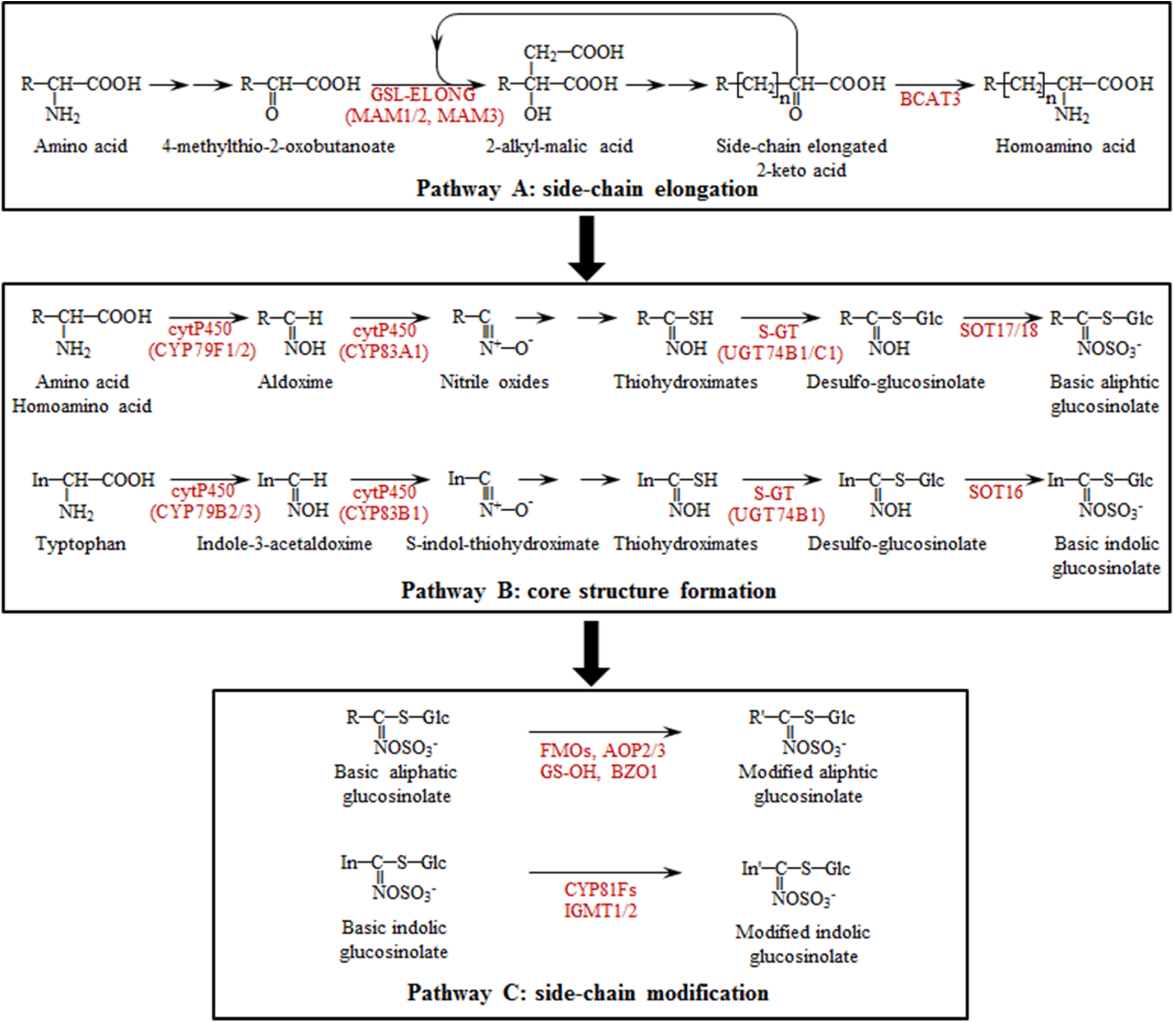
Glucosinolate biosynthetic pathways in Brassicaceae. Arrows between compounds represent the number of putative enzymatic reactions. For simplicity, only genes discussed in the text have been included. The figure is constructed following several publications [17–19].

Plants are able to produce a diverse array of compounds that contribute to defense against herbivores and pathogens [7]. This includes regulatory compounds such as jasmonic acid, salicylic acid and ethylene as well as defensive compounds such as glucosinolates (GSLs). The regulatory and defense compounds have to coordinate with each other to jointly participate in the plant defense system [8]. As the key components of defense metabolites, GSLs and their breakdown products have been identified to have potential antiherbivore [9–11] and antimicrobial properties [12–14]. The chemical structure of GSLs comprises a common core together with a variable side chain [15]. According to the type of amino acid precursor from which the side chain is derived, GSLs are classified into three classes: aliphatic, benzenic or indolic GSLs (For GSL abbreviations, see Fig 1) [16]. The GSL biosynthesis pathway in the order Brassicales has been reported to contain three steps: side-chain elongation, core-structure formation and side-chain modification (Fig 2) [17–19].

*B*. *rapa*, *B*. *oleracea* and *B*. *napus* are three important Brassica crops and shared a common ancestor with *Arabidopsis thaliana* [20–23]. The identification of the genes in Arabidopsis GSL biosynthesis allows for the extension of this knowledge to the Brassica species [19]. In *B*. *oleracea*, two genes (*BoGSL-ELONG* and *BoGSL-PRO*) are involved in side chain elongation, and one gene (*GSL-ALK*) is involved in side chain modification [24, 25]. *BoGSL-ELONG* was cloned based on the sequence information of the *MAM* (methylthioalkylmalate synthase) family genes in Arabidopsis, and was functionally characterized using an RNA interference (RNAi) approach in *B*. *napus* [24, 26]. The results suggested that *BoGSL-ELONG* is involved in 4C and 5C aliphatic GSL biosynthesis in Brassicaceae. Additionally, *BoGSL-PRO* that controls 3C GSL biosynthesis in *B*. *oleracea* was also coloned using a comparative analysis of the Arabidopsis *MAM* family genes [25, 27]. In *B*. *rapa*, the *MAM* gene family orthologous to the *MAM* genes in Arabidopsis controls the step of side chain elongation in GSL biosynthesis [28, 29]. In GSL core-structure formation, aldoximes are oxidized into either nitrile oxides or aci-nitro compounds by *AtCYP83A1* and *AtCYP83B1*, the two genes of cytochrome P450 family. The former converts aliphatic aldoximes to thiohydroximates, and the latter metabolizes both Trp-derived and Phe-derived acetaldoximes [30–34]. In *B*. *rapa*, two *CYP83A1* genes named *BrCYP83A1-1* and *BrCYP83A1-2* were respectively cloned from pak choi, but no functional analysis was conducted with the two genes [35]. Thiohydroximates are in turn S-glucosylated by glucosyltransferases of the UGT74 family, UGT74B1 and UGT74C1, forming desulfoglucosinolates in Arabidopsis [36–38]. Insertional *ugt74b1* mutant lines showed significantly decreased aliphatic and indolic GSLs [36]. Another member, *UGT74C1*, can complement the phenotypes and chemotypes of *ugt74b1* mutant and shows thiohydroximate UGT activity *in vitro*, suggesting that *UGT74C1* is an accessory enzyme in GSL biosynthesis with a potential function during plant’s adaptation to environmental challenges [37, 38].

Aliphatic GSLs are important in resistance to chewing insects and some adapted pathogens [14, 39–42]. The chain length of aliphatic GSLs determined by the *GSL-ELONG* locus is a critical factor for the resistance to both diamondback moth (Plutella xylostella) [43] and two specialist aphid species [44]. A recent study showed that *AtCYP83A1* is very important for aliphatic GSL metabolism for the resistance to the *B*. *cinerea* in Arabidopsis, but the loss-function mutant *cyp83a1* showed a dramatically reduced parasitic growth of the biotrophic powdery mildew fungus *Erysiphe cruciferarum (E*. *cruciferarum)* [40]. Interestingly, a *myb28myb29* double mutant, which totally lacks aliphatic GSLs, showed a wild-type level of susceptibility to *E*. *cruciferarum*. The authors explained that the *cyp83a1* mutant might have a reduced supply of the fungus with inductive signals from the host and an accumulation of potentially fungitoxic metabolites [2, 40, 45]. This result suggests that aliphatic GSLs display different effects in the resistance to biotrophic and necrotrophic pathogens. Indolic GSLs play a predominant role in defense against non-host pathogens and oviposition by specialized lepidopteran moths [9, 11, 13, 46–48]. The plant lacking indolic GSLs shows a higher suscceptivity to pathogens, especially the necrotrophic fungi such as *Alternaria brassicicola* [49], *Plectospaerella cucumerina* [50], *S*. *sclerotiorum* [48] and *B*. *cinere*a [51]. In the Brassicaceae, the levels of indolic GSLs in *B*. *napus* are positively correlated with the resistance to *S*. *sclerotiorum* [52–54].Despite the obvious importance of GSLs in the resistance against various pathogen infections, there have been few reports on the use of GSL metabolism genes to enhance the stress resistance in *B*. *napus* by a transgenic approach. Importantly, most modern varieties of *B*. *napus* are referred to as ‘double lows’ as they have seeds with low GSL and erucic acid. The reduction of seed GSL is thought to be associated with a concomitant decrease in GSL production in leaves [55], resulting in decreased plant disease resistance [56].

The current study was aimed to explore the possibility of enhancement in the resistance to fungal diseases in *B*. *napus* based on the understanding of the biological function of GSLs as revealed in model plant Arabidopsis previously. We found that the expression of three GSL biosynthesis genes (*BnMAM1*, *BnCYP83A1* and *BnUGT74B1*) in *B*. *napus* were highly responsive to either or both of *S*. *sclerotiorum* or *B*. *cinerea* infection. Transgenic *B*. *napus* plants overexpressing these three genes individually showed enhanced foliar GSL levels. Subsequently, the overexpressing lines were inoculated with *S*. *sclerotiorum* and *B*. *cinerea*, to investigate the effects of the three genes on enhancing the pathogen resistance of *B*. *napus*. Our results indicate a new and important method to strengthen the resistance against *S*. *sclerotiorum* and *B*. *cinerea* by overexpressing GSL biosynthesis genes in *B*. *napus*.

## Materials and Methods

### Plant materials and growth conditions

*B*. *napus* plants were planted in the isolated nursery field (Huazhong Agriculture University experimental farm, Wuhan, China) and green house (University of California Davis, Davis, California, USA). The field trial and green house did not require any specific permits as the nursery was set up for this type study. For some pre-test experiments, plants were grown in a greenhouse at 23/18°C (day/night) under a 13-h illumination time with a light density of 230–300 μEm^-2^s^-1^.

### Gene cloning, sequence alignment and phylogenetic analysis

Total RNA was extracted from the leaves of *B*. *napus* using the Plant Total RNA Extraction Kit (Biotake). For each sample, 2 μg of total RNA was used for reverse transcription with TransScript First-Strand cDNA Synthesis Super Mix (TransGen). *BnMAM1*, *BnCYP83A1* and *BnUGT74B1* were cloned from a cDNA library of *B*. *napus cv*. *Jia2201* and the primers for cloning were designed based on the sequence information of Arabidopsis *AtMAM1*, *AtCYP83A1* and *AtUGT74B1*, respectively. All cloning primers are listed in S1 Table. Sequence alignment was performed using CLUSTALX1.83 [57], and phylogenetic analysis was applied to the alignment results. The similarity of the predicted protein sequences were compared based on a BLOSUM62 matrix [58].

### Plasmid construction and plant transformation

Full-length *BnMAM1*, *BnCYP83A1* and *BnUGT74B1* cDNA were amplified with the primers YYp01 and YYp02, YYp19 and YYp20B, YYp07 and YYp08, respectively, using the cDNA from *B*.*napus* (cultivar Jia 2201) leaves as a template. The fragments were cloned to pMD18-T (Takara) and sequenced. The confirmed fragments were then digested with *Xba*I and *Sac*I and sub-cloned into the corresponding sites of pBI121 with a CaMV 35S promoter. The constructs for plant transformation were introduced into *Agrobacterium tumefaciens* GV3101 by electro-transformation. *B*. *napus* (cultivar Jia 572) plants were transformed according to the method as described by Zhou [59]. Putative transformants (T_0_) were transferred to soil. DNA was isolated from young leaves and used to determine the presence of the transgene by PCR using CaMV 35S promoter-specific forward primer (35S-5) and gene-specific reverse primer. Primers used in this study are listed in S1 Table.

### DNA preparation and Southern blot analyses

Genomic DNA was isolated from young leaves of each transgenic line by CTAB extraction protocols [60]. For DNA blotting analysis, 20 μg of *B*.*napus* genomic DNA was digested with *EcoRI* (New England Biolabs) and separated on 0.8% agarose gel for each line. After electrophoresis, the digested DNA was transferred onto a Hybond N^+^ nylon membrane (Amersham). For hybridization, a conserved 522 bp ^32^P-labeled NPTII 3’-terminal sequence was used as a probe. The membrane was hybridized for 24 h at 55°C, and then washed twice with a solution of 0.1 × SSC and 0.1% SDS at 65°Cfor 20 min. The hybridized membrane was scanned with a FUJI FLA-9000 image analysis system (Fujifilm) following the manufacturer’s instructions as previously described [61].

### Glucosinolate Analysis

7 weeks after sowing when the plants had six true leaves and before bolting, the fourth leaf from the bottom of each plant was collected and stored in 90% methanol to inhibit enzymatic breakdown of chemical compounds and to prepare for the extraction. In total, 6 plants were sampled for each line. At maturity, seeds were harvested from the same 6 plants and were fully dried. 2-Propenyl/allyl GSL (Sinigrin, Sigma-Aldrich) was used as the internal standard. GSLs were extracted from the leaves and analyzed by high-performance liquid chromatography (HPLC) according to previously described methods [62] with some modifications. One leaf was harvested into a 50-ml tube with 15 ml of 90% methanol and fifteen 3-mm ball bearings, or 200 mg of dried seeds were added into a 2-ml tube with 1 ml of 90% methanol and two 3-mm ball bearings. Then the samples were ground into fine powder in a paint shaker by high-speed agitation. The final volume of GSL extract was 500 μl, and 10μl extract was measured by HPLC. The entire experiment was replicated three times.

For data analysis, GSL contents were analyzed via ANOVA using a general linear model within the R software package (x64 3.1.2) [63]. For all overexpressing lines, each of them was tested for altered GSL content in an individual ANOVA against WT. All three independent experiments were combined and an experiment term was included in the model to test for effects.

### Plant inoculation

*Sclerotinia sclerotiorum* (Ss-1) and *Botrytis cinerea* (Bc- Canola-3) cultured on PDA were provided by Prof. Guoqing Li (State Key Laboratory of Agricultural Microbiology, Huazhong Agricultural University). The PCR-positive plants and controls (7-week-old) were used for inoculation. Inoculation of the detached leaves was performed as described previously [5]. The experiment was in a randomized complete block design and was repeated three times. For each replicate, we sampled more than 12 leaves with each leaf coming from an individual plant. At 48h after inoculation of *S*. *sclerotiorum* or 96h after inoculation of *B*. *cinerea*, the lesion size (LS) was measured and calculated with the formula LS = (a+b)/2, where a and b represent the long and short diameters of lesions, respectively.

### Gene expression analysis

Total RNA was prepared from the sampled tissues with TRIZOL reagent (Invitrogen). For each sample, 5 μg RNA was treated with 10 U DNase I (New England Biolabs) to remove the residual DNA, and was then used for reverse transcription reaction with the TransScript First-Strand cDNA Synthesis Super Mix (TransGen). qRT-PCR was performed with three technical replicates on a Bio-Rad CFX96 Real-Time system (Bio-Rad) and DBI Bioscience Bestar-Real Time PCR Master Mix kit, following the manufacturer’s instructions (DBI Bioscience) as previously described [64]. The data were analyzed with LINREG, as described by Ramakers, Ruijter [65]. The experiment was repeated using three independent biological replicates. Primers used in this study are listed in S1 Table.

## Results

### Response of *BnMAM1*, *BnCYP83A1* and *BnUGT74B1* to *S*. *sclerotiorum* and *B*. *cinerea* infection in *B*. *napus*

**Fig 3:**
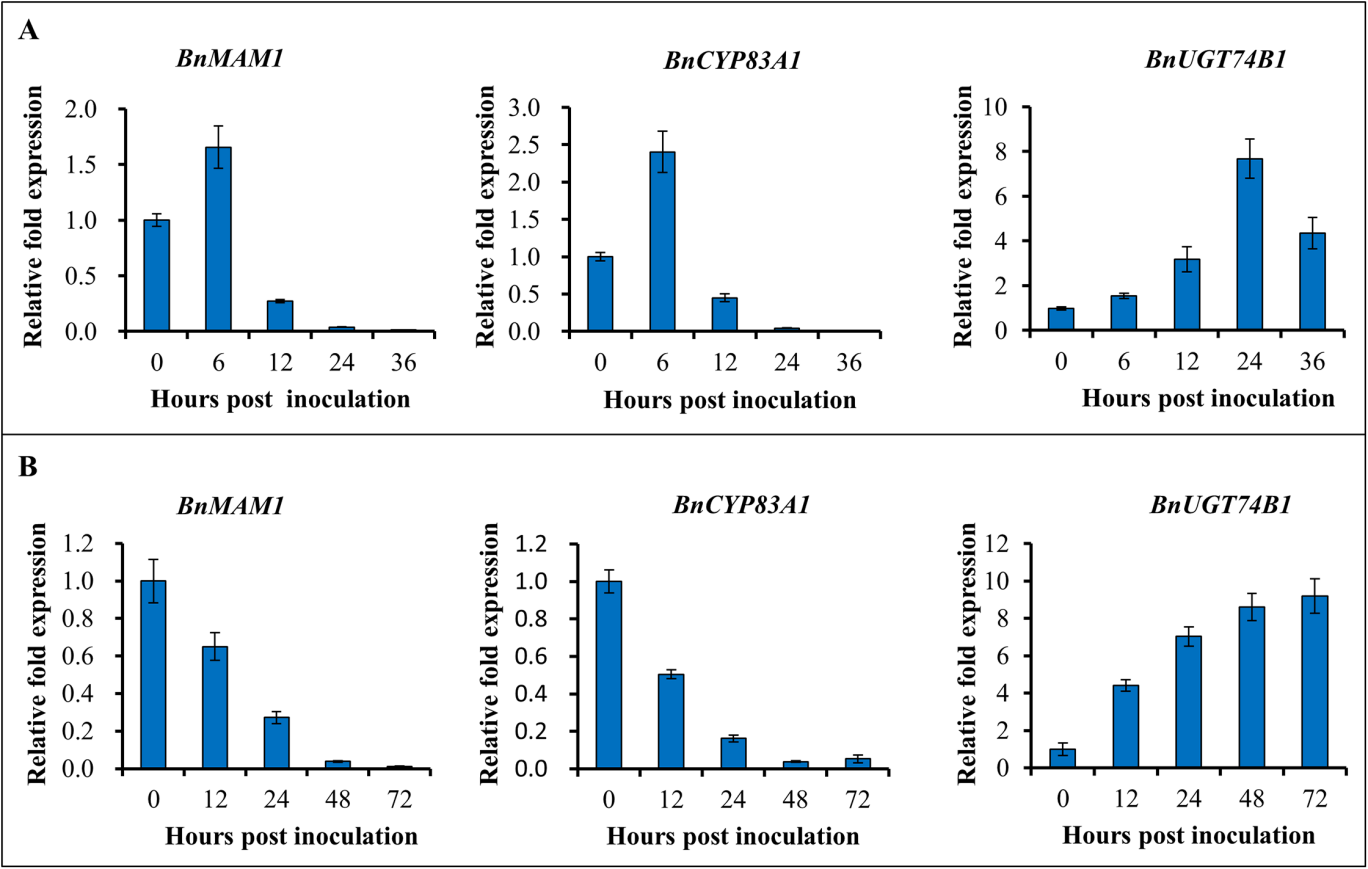
Response of *BnMAM1*, *BnCYP83A1* and *BnUGT74B1* to *Sclerotinia sclerotiorum* and *Botrytis cinerea* infection. Relative expression levels of *BnMAM1*, *BnCYP83A1* and *BnUGT74B1* in *Brassica napus* were determined by qRT-PCR at 0, 6, 12, 24 and 36 h post *S*. *sclerotiorum* inoculation (A) and 0, 12, 24, 48 and 72 h post *B*. *cinerea* inoculation (B). The expression levels were relative to no inoculation (0 h) and quantified by qRT-PCR. Values are means of three replicates. Each bar represents means ± SE.

Previous studies in Arabidopsis showed that *AtMAM1*, *AtCYP83A1*, *AtUGT74B1* are genes located at the key points in the GSL biosynthesis pathway [17–19], and are related to both aliphatic and indolic GSL synthesis as revealed by mutant analyses [30, 36, 66]. However, if the genes homologues in *B*.*napus* are responsive to fungal pathogens infection was not clear. To determine whether *BnMAM1*, *BnCYP83A1* and *BnUGT74B1* function in the defense responses to *S*. *sclerotiorum* and *B*. *cinerea*, we used wild-type (WT) *B*. *napus* to measure the expression of the three genes in response to pathogen infection by qRT-PCR (Fig 3). The results show that following inoculation with *S*. *sclerotiorum*, the accumulation of *BnMAM1* and *BnCYP83A1* transcripts is induced rapidly and reached a peak within 6 hpi. But this induction was transient, and the expression levels of the two genes decreased to non-infection level after 24 hpi (Fig 3A). In contrast, the expression of *BnUGT74B1* was slowly induced before 12 hpi and was rapidly induced thereafter until 24 hpi when it reached the maximum, and then it was weakly down-regulated till 36 hpi. These results suggest that both aliphatic and indolic GSLs biosynthesis genes are responsive to *S*. *sclerotiorum* infection.

Interestingly, the expression patterns of the three genes in response to *B*. *cinerea* inoculation were different from those in response to *S*. *sclerotiorum* inoculation. After inoculation with *B*. *cinerea*, the expression of *BnMAM1* and *BnCYP83A1* was continuously down-regulated from the beginning (Fig 3B); conversely, the expression of *BnUGT74B1* was up-regulated from the beginning to the end. These results suggest that the genes involved in indolic GSL biosynthesis are more active than those in aliphatic GSL biosynthesis in response to the infection by *B*. *cinerea*.

### Overexpression of *BnMAM1*, *BnCYP83A1* and *BnUGT74B1* in *B*. *napus*

**Fig 4:**
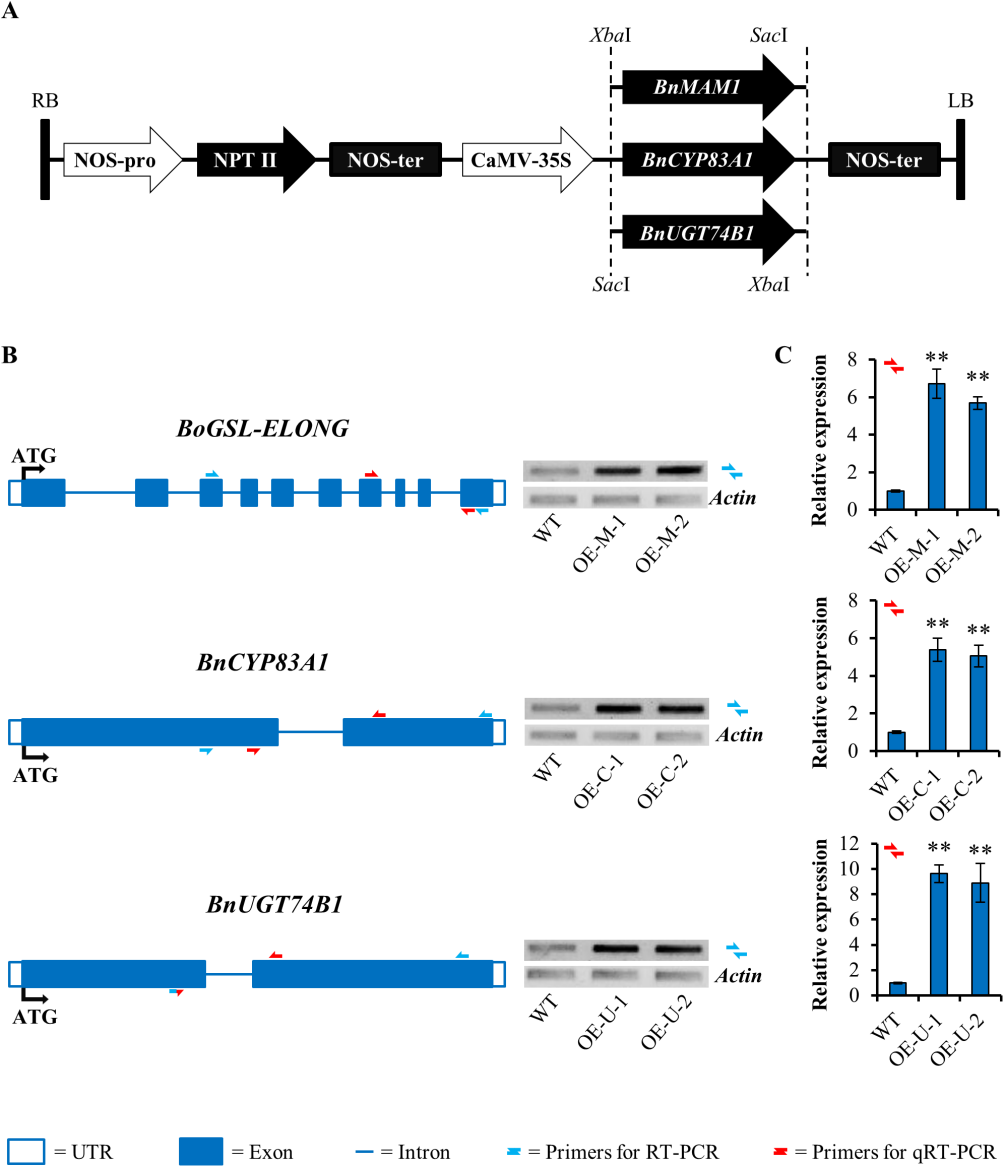
Characterization of *BnMAM1*-, *BnCYP83A1*- and *BnUGT74B1*-overexpressing lines in *Brassica napus*. (A) Diagram of the plasmids used in this study. pBI121 contain *BnMAM1*, *BnCYP83A1* or *BnUGT74BA1* cDNAs, respectively. RB, right border; NOS-pro, nopaline synthase gene promoter; NPTII, coding region of neomycin phosphotransferase II gene; CaMV-35S, cauliflower mosaic virus 35S promoter; NOS-ter, nopaline synthase gene terminator; LB, left border. (B) Molecular characterization of *BnMAM1*-, *BnCYP83A1*- and *BnUGT74BA1*-overexpressing lines. Structure of the *BnMAM1*, *BnCYP83A1* or *BnUGT74BA1* genes including exon and intron boundaries. Accumulation of *BnMAM1*, *BnCYP83A1* or *BnUGT74BA1* mRNA in WT (untransformed wild-type control) and corresponding two independent transgenic T2 lines were measured by RT-PCR on 7-week-old leaves. Actin 7 (*ACT7*, *Brassica napus*) gene expression was used as a constitutive control. Primers used for this study are indicated as solid black arrows (See primers in S1 Table). (C) Accumulation of *BnMAM1*, *BnCYP83A1* or *BnUGT74BA1* mRNA in 7-week-old leaves of WT control and corresponding overexpressing lines. The expression level of Actin 7 (*ACT7*, *Brassica napus*) was used as a constitutive control. Values are the means and SE of three replicates performed on cDNA dilutions obtained from three independent mRNA extractions. The significant differences is shown as ** (*P* < 0.05) on the bar. Primers used for this study are indicated as hollow white arrows (See primers in S1 Table).

To explore the possibility if the resistance to *S*. *sclerotiorum* and *B*. *cinerea* could be modified by manipulation of GSL levels in host plants through overexpressing *BnMAM1*, *BnCYP83A1* and *BnUGT74B1*, we cloned the three genes from the cDNA library of *B*. *napus cv*. *Jia2201* (a high-GSL cultivar) by a homology cloning approach (S1–S3 Figs). Subsequently, the cloned genes were inserted behind the CaMV 35S promoter respectively in the vector pBI121 (Fig 4A), and the constructs were separately transformed into *B*. *napus cv*. *Jia572*, a low-GSL cultivar. More than thirty lines for each construct were produced. Kanamycin and PCR were used to screen each transgenic line (Fig 4B). Southern blot analysis was performed using a probe specific to the *NPTII* fragment (S4 Fig). Except for a few plants that showed chlorotic leaves and that did not survive to maturity, most transgenic plants did not show phenotypic changes during any developmental stage. Based on the results of southern blot analysis and qRT-PCR analysis, two independent transgenic lines with higher expression of the corresponding gene (Fig 4C) were chosen for each construct, and were used for further analysis.

### Glucosinolate content analysis

To investigate the effect of overexpression of the three GSL biosynthesis genes, we first compared the leaf GSL profiles of each 7-week-old T_2_ transgenic line and the controls (untransformed wild-type). The GSL contents were independently measured in the leaves of more than 6 plants for each T_2_ transgenic line, and three aliphatic and three indolic GSLs were detectable (Table 1). 2OH3B GSL, 3-butenyl GSL and 4-pentenyl GSL all belong to aliphatic GSLs. I3M, 4OH-I3M and 4MO-I3M are all indolic GSLs. Compared with in the wild type (WT) plants, in the *BnMAM1*-overexpressing lines, two 4-carbon (C4) side-chain aliphatic GSLs (2OH3B and 3-Butenyl GSL) accumulated to 1.7 and 1.5 fold higher levels respectively, while the content of another 5-carbon (C5) aliphatic GSL (4-Pentenyl GSL) was similar to that of WT. The contents of all the indolic GSLs were the same as those of WT. *BnCYP83A1*-overexpressing lines showed a similar GSL profile to *BnMAM1-*overexpressing lines; their 2OH3B and 3-Butenyl GSL contents were increased to 2.2 and 1.8 fold higher than WT. In *BnCYP83A1*-overexpressing lines, there were the same contents of both 4-Pentenyl and all the indolic GSLs as WT. In all the transgenic lines, only the *BnUGT74B1-*overexpressing lines showed increased indolic GSLs content, among which I3M GSL was increased to 1.5 fold that of WT, while 4OH-I3M and 4MO-I3M GSL showed no difference. *BnUGT74B1*-overexpressing lines also lead to increased 2OH3B and 3-Butenyl GSL content, while the content of 4-Pentenyl GSL was not changed when compared with those of WT. Interestingly, the GSLs accumulation in seeds is a little different from in leaves. In all transgenic lines examined, only one aliphatic GSL (2OH3B GSL) content was increased compared with WT plants (S2 Table), and other aliphatic GSLs (3-Butenyl, 4-Pentenyl and 5MSOP GSL) were similar to WT. For indolic GSL, only the *BnUGT74B1*-overexpressing lines showed increased I3M content compared with WT.

**Table 1:** GSL contents (nmol/g) in 7-week-old leaves of *BnMAM1*, *BnCYP83A1* and *BnUGT74B1* overexpressing T_2_ lines.

GSL^a^	Wild type	*BnMAM1* overexpressing lines				*BnCYP83A1* overexpressing lines				*BnUGT74B1* overexpressing lines			
	WT	OE-M-1	*P* ^b^	OE-M-2	*P*	OE-C-1	*P*	OE-C-2	*P*	OE-U-1	*P*	OE-U-2	*P*
2OH3B	13.70±2.72	24.05±2.81	0.017	25.10±3.75	0.018	35.04±10.03	0.033	29.88±3.95	0.002	24.61±5.61	0.025	32.75±7.61	0.022
3-Butenyl	12.51±1.64	20.52±2.18	0.006	17.49±1.06	0.026	26.02±3.86	0.002	19.61±2.86	0.031	28.11±5.53	0.009	26.03±4.26	0.005
4-Pentenyl	20.32±4.96	24.44±4.97	NS^c^	25.75±5.59	NS	23.24±5.14	NS	23.99±2.41	NS	26.07±6.23	NS	25.48±5.29	NS
I3M	167.58±17.78	165.34±18.52	NS	150.35±17.74	NS	166.31±18.23	NS	156.27±20.96	NS	245.31±32.94	0.013	240.22±31.04	0.028
4OH-I3M	5.32±0.53	6.45±0.73	NS	5.27±0.53	NS	7.43±1.33	NS	5.88±0.83	NS	7.43±0.87	NS	7.18±0.92	NS
4MO-I3M	11.47±0.82	11.25±1.15	NS	12.04±0.98	NS	12.06±1.00	NS	12.83±1.27	NS	13.39±0.98	NS	13.68±1.20	NS
Total Aliphatic	46.52±8.59	69.01±8.13	0.041	68.33±7.83	0.043	84.31±16.07	0.038	73.48±7.99	0.035	78.79±15.72	0.047	84.26±16.04	0.043
Total Indolic	184.37±18.03	183.05±18.57	NS	167.65±18.17	NS	185.8±18.40	NS	174.98±22.45	NS	266.13±34.17	0.04	261.07±32.00	0.042
Total	230.88±20.91	252.06±24.44	NS	235.99±21.58	NS	270.11±27.45	NS	248.46±27.88	NS	344.92±45.89	0.028	345.34±38.56	0.013

The data (means ±SE and *P* value, n = 18) were collected from three independent experiments and were analyzed via ANOVA.

^a^For GSL abbreviations, see Fig 1.

^b^ *P* value for GSL differences between the overexpression line and WT as determined by ANOVA.

^c^Not a significant *P* value (*P* > 0.05).

### Inoculation with *S*. *sclerotiorum and B*. *cinerea*

**Fig 5:**
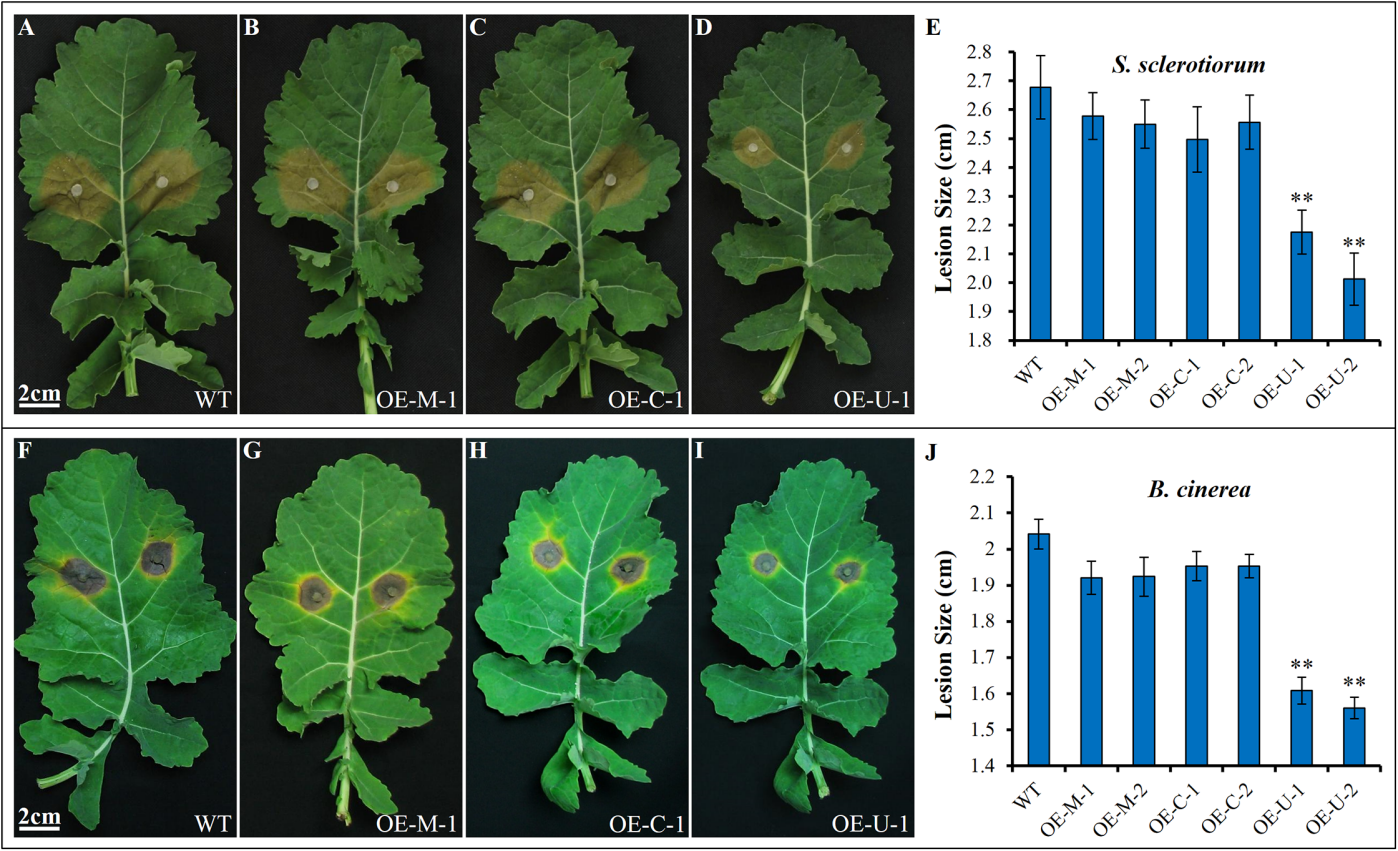
Resistance of *BnMAM1*, *BnCYP83A1* and *BnUGT74B1* overexpressing T_2_ plants to *S*. *sclerotiorum* and *B*. *cinerea*. (A-D) Disease responses of inoculated plants with *S*. *sclerotiorum* at 48 h post-inoculation (hpi). (E) Lesion sizes of leaves inoculated with *S*. *sclerotiorum*, which were measured for the first 48 h after inoculation. Means and SE are shown (n ≥ 12). The significant differences is shown as ** (P < 0.05) using t-tests. OE- M-1 and OE-M-2 are transgenic lines for *BnMAM1*, OE- C-1 and OE-C-2 are transgenic lines for *BnCYP83A1*, OE- U-1 and OE-U-2 are transgenic lines for *BnUGT74B1*. WT, untransformed wild-type control. (F-I) Disease responses of inoculated plants with *B*. *cinerea* at 96 hpi. (J) Lesion sizes of leaves inoculated with *Botrytis cinerea*, which were measured at 96 hpi. Means and SE are shown (n ≥ 12). The significant differences is shown as ** (P < 0.05) using t-tests.

To determine the effects of overexpressing the three genes on the resistance to *S*. *sclerotiorum*, positive T_2_ transgenic plants were selected by PCR. Leaves from these PCR-positive plants and the controls (WT) at the six-true-leaf stage were inoculated with mycelial plugs of *S*. *sclerotiorum* (Fig 5A–5D) and *B*. *cinerea* (Fig 5F–5I). For the *S*. *sclerotiorum* (Ss-1) inoculum, the results indicate that only *BnUGT74B1*-overexpressing lines developed less severe disease symptoms and less tissue damage than the control, while no significant difference was observed between *BnMAM1-* and *BnCYP83A1*-overexpressing lines and the control at 48 h post-inoculation (hpi) (Fig 5E). Investigation of disease progression shows that soft-rotting necrosis occurred as early as 16 hpi, and we counted the leaves with necrosis. The results show that more than 90% of *BnMAM1*- and *BnCYP83A1*-overexpressing plants and the control were infected, but necrosis was observed in less than 10% of *BnUGT74B1*-overexpressing plants (Table 2). Necrosis was delayed in most of the *BnUGT74B1*-overexpressing plants until 24 hpi.

**Table 2:** The morbidity rate of *S*. *sclerotiorum* infection at early stage.

	Wild type	*BnMAM1*		*BnCYP83A1*		*BnUGT74B1*	
	WT	OE-M-1	OE-M-2	OE-C-1	OE-C-42	OE-U-1	OE-U-2
16 hpi	46	47	37	37	42	3	4
Total	49	51	40	39	46	41	43
Ratio	93.88%	92.16%	92.50%	94.87%	91.30%	7.32%	9.30%

The ratio of the number of inoculation sites showing necrosis to the total number of inoculation sites was expressed as early stage morbidity. The lines OE-M-1 and OE-M1-2 are transgenic for *BnMAM1*. The lines OE-C-1 and OE-C-2 are transgenic for *BnCYP83A1*. The lines OE-U-1 and OE-U-2 are transgenic for *BnUGT74B1*. WT, untransformed wild-type control.

We also inoculated the transgenic lines with *B*. *cinerea* (Bc- Canola-3), which is closely related *S*. *sclerotiorum* as a necrotrophic plant pathogenic fungus. The results are similar to those of the inoculation of *S*. *sclerotiorum*. Only *BnUGT74B1*-overexpressing lines showed smaller lesion areas than the control at 96 hpi (Fig 5J), and the infection rate was significantly lower than the control at 32hpi, when the gray mold diseases just occurred in other transgenic lines and the control (Table 3). All the results of inoculation with *S*. *sclerotiorum* and *B*. *cinerea* suggest that the overexpression of *BnUGT74B1* could significantly inhibit or delay the necrotic diseases caused by *S*. *sclerotiorum* and *B*. *cinerea*.

**Table 3:** The morbidity rate of *B*. *cinerea* infection at early stage.

	Wild type	*BnMAM1*		*BnCYP83A1*		*BnUGT74B1*	
	WT	OE-M-1	OE-M-2	OE-C-1	OE-C-42	OE-U-1	OE-U-2
32 hpi	23	38	34	33	36	2	3
Total	24	41	36	36	40	25	25
Ratio	95.83%	92.68%	94.44%	91.67%	90.00%	8.00%	12.00%

The ratio of the number of inoculation sites showing necrosis to the total number of inoculation sites was expressed as early stage morbidity. The lines OE-M-1 and OE-M1-2 are transgenic for *BnMAM1*. The lines OE-C-1 and OE-C-2 are transgenic for *BnCYP83A1*. The lines OE-U-1 and OE-U-2 are transgenic for *BnUGT74B1*. WT, untransformed wild-type control

## Discussion

Over the past two decades, almost all genes involved in the GSL biosynthesis pathway have been identified and characterized in model plant *Arabidopsis thaliana* [19]. Loss-of-function and gain-of-function studies have demonstrated that aliphatic and indolic GSLs play important roles in plant defence, but little direct evidence has been found for the specific GSL biosynthesis genes from *B*. *napus* in defense against the pathogens *S*. *sclerotiorum* and *B*. *cinerea*. Our study provides new data that *BnMAM1*, *BnCYP83A1* and *BnUGT74B1* are all highly responsive to *S*. *sclerotiorum* and *B*. *cinerea* infection, and overexpression of these three genes led to marked increase in either or both of aliphatic and indolic GSLs levels in leaves. All three genes overexpression significantly increased two aliphatic GSLs levels (2OH3B and 3-Butenyl GSLs) compared with the wild type control, but only *BnUGT74B1* overexpression increased indolic GSL content (I3M GSL) (Table 1). We further find that only *BnUGT74B1* overexpression enhances the resistance to *S*. *sclerotiorum* and *B*. *cinerea* while *BnMAM1-* and *BnCYP83A1*-overexpressing lines show similar disease symptoms and tissue damage to the wild type control (Fig 5, Tables 2 and 3).

In Brassicaceae, *BoGSL-ELONG* and *BoGSL-PRO* involved in side chain elongation have been identified and characterized in *B*. *oleracea*. The former is involved in the biosynthesis of four-carbon (4C) GSLs, whereas the latter is involved in the biosynthesis of three-carbon (3C) GSLs. [24, 25, 67]. *MAM* gene silencing in *B*. *napus* significantly induced the production of 3C GSLs, while the contents of 4C and two 5C side-chain GSLs were decreased [26]. Similar glucosinolate contents were detected by silencing *BnGSL-ALK* gene families in *B*. *napus* [68]. In our study, two 4C side-chain aliphatic GSLs (2OH3B and 3-Butenyl GSLs) contents were increased by overexpression of *BnMAM1* compared with the wild type control (Table 1), while the level of 5C side-chain GSL (4-Pentenyl GSL) was similar to wild type control. These results indicate that the copy of *BnMAM1* used in this study may only catalyze the elongation of 3C to 4C GSLs, and there may be other copies responsible for production of 5C GSLs.

When the transgenic lines were inoculated with *S*. *sclerotiorum*, *BnMAM1* and *BnCYP83A1* showed the same transcription profile: they were up-regulated at the early stage (6 hpi) and were rapidly down-regulated thereafter (Fig 3A). By contrast, the expression of *BnUGT74B1* was continuously induced especially after 12 hpi while it was only slightly induced before 12 hpi. These results suggest that aliphatic GSL biosynthesis pathway was induced for defending against *S*. *sclerotiorum* infection at the early stage while the *BnMAM1-* and *BnCYP83A1*-overexpressing lines show similar disease symptoms and tissue damage to the wild type control. By contrast, with the inoculation of *B*. *cinerea*, the expression of *BnUGT74B1* was increased during the whole disease procession, but the expression of *BnMAM1* and *BnCYP83A1* were down-regulated from the beginning (Fig 3B). These results show that indolic GSL play an important role in defense against *B*. *cinerea* infection, while the aliphatic GSLs contribute little to the defense. The degradation products indolic GSLs, especially I3M and 4MOI3M, are very important defense compounds to biotic and abiotic stresses [10, 12, 13, 48, 69, 70]. In our study, overexpression of *BnUGT74B1* not only significantly increased the level of I3M GSL in transgenic plants but also significantly enhances the resistance of the transgenic plants to *S*. *sclerotiorum* and *B*. *cinerea* (Fig 5). Although it seems that aliphatic GSL do not play an important role in the defense against necrotrophic *S*. *sclerotiorum* and *B*. *cinerea* pathogens, but it could provide the resistance to other pests and diseases, such as chewing insects and some adapted pathogens [14, 39–42]. So far, no immune or highly resistant germplasm for *S*. *sclerotiorum* and *B*. *cinerea* in *B*. *napus* has been reported, and few germplsms of host resistance to the pathogen are available to breeders [1]. The resistance to the pathogens in plants exhibits mainly quantitative variation controlled by quantitative genes [5]. Many factors including GSLs content in plants may contribute to the differences in resistance among different genotypes [12]. Currently the genetic improvement of resistances to these two fungal pathogens still heavily relies on the accumulation of such quantitative resistance [5]. In this study, such kind of the resistance by overexpressing GSL biosynthesis gene, although only as significant as quantitative resistance, is still valuable for the breeding for necrotic fungal infection.

As plant matured, GSLs are transported from vegetative tissues to reproductive tissues [55, 71, 72]. In our study, the results of seeds GSLs showed that the GSLs accumulations in leaf and seed are different and the increasing content of leaves does not necessarily lead to the increase in seeds for individual GSLs. Such a difference is likely due to GSL transport mechanism [73] and secondary modifications [17–19]. Therefore it is possible that we can silence the GSL transporters, such as GTR1 and GTR2 [73], to maintain higher levels of GSLs in vegetative tissues while to restrict a low level in the seed. However, the molecular mechanisms of host defense to *S*. *sclerotiorum* remain poorly understood in the GSL–*S*. *sclerotiorum* interaction, which restricts the engineering of resistance by transgenic approaches and still need further elucidate.

In general, the resistance to two necrotrophic fungi *S*. *sclerotiorum* and *B*. *cinerea* was enhanced in transgenic *B*. *napus* plants by overexpressing *BnUGT74B1*, which is complementary to the positive effects observed in a study on indolic GSL [12–14, 48, 51]. The results may facilitate the understanding of the mechanisms underlying the resistance to *S*. *sclerotiorum* and *B*. *cinerea*, which may provide clues to the development of effective strategies for controlling the diseases caused by *S*. *sclerotiorum* and *B*. *cinerea*.

## Supporting Information

S1 Table List of primers used in constructs and assays. (DOCX)

S2 Table GSL contents (μmol/g) of seeds from *BnMAM1*, *BnCYP83A1* and *BnUGT74B1* overexpressing T_2_ lines. (DOCX)

S1 Fig Alignment of the amino acid sequence of AtMAM1 and BnMAM1. (TIF)

S2 Fig Alignment of the amino acid sequence of AtCYP83A1and BnCYP83A1. (TIF)

S3 Fig Alignment of the amino acid sequence of AtUGT74B1and BnUGT74B1. (TIF)

S4 Fig Southern blot analysis of *EcoRI* -digested genomic DNA of each transgenic lines. The copy number of each transgene was estimated based on the number of the bands seen on Southern blots. The genomic DNA was digested with *EcoRI* and a conserved 522 bp ^32^P-labeled NPTII 3’-terminal sequence was used as a probe. WT, untransformed wild-type control. OE- M-1 and OE-M-2 are transgenic lines for *BnMAM1*, OE- C-1 and OE-C-2 are transgenic lines for *BnCYP83A1*, OE- U-1 and OE-U-2 are transgenic lines for *BnUGT74B1*.
(TIF)

## References

[1] Amselem J, Cuomo CA, van Kan JA, Viaud M, Benito EP, Couloux A, et al Genomic analysis of the necrotrophic fungal pathogens *Sclerotinia sclerotiorum* and *Botrytis cinerea* . PLoS genetics. 2011;7(8):e1002230 10.1371/journal.pgen.1002230 21876677 PMC3158057

[2] Rowe HC, Walley JW, Corwin J, Chan EK-F, Dehesh K, Kliebenstein DJ. Deficiencies in jasmonate-mediated plant defense reveal quantitative variation in *Botrytis cinerea* pathogenesis. PLoS pathogens. 2010;6(4):e1000861 10.1371/journal.ppat.1000861 20419157 PMC2855333

[3] Bolton MD, Thomma BP, Nelson BD. *Sclerotinia sclerotiorum* (Lib.) de Bary: biology and molecular traits of a cosmopolitan pathogen. Molecular Plant Pathology. 2006;7(1):1–16. 10.1111/j.1364-3703.2005.00316.x 20507424

[4] Williamson B, Tudzynski B, Tudzynski P, van Kan JA. *Botrytis cinerea*: the cause of grey mould disease. Molecular Plant Pathology. 2007;8(5):561–580. 10.1111/j.1364-3703.2007.00417.x 20507522

[5] Wu J, Cai G, Tu J, Li L, Liu S, Luo X, et al Identification of QTLs for resistance to Sclerotinia stem rot and *BnaC*. *IGMT5*. a as a candidate gene of the major resistant QTL *SRC6* in *Brassica napus* . PloS one. 2013;8(7):e67740 10.1371/journal.pone.0067740 23844081 PMC3699613

[6] Oliver RP, Solomon PS. New developments in pathogenicity and virulence of necrotrophs. Current opinion in plant biology. 2010;13(4):415–419. 20684067

[7] Kliebenstein DJ. Plant defense compounds: systems approaches to metabolic analysis. Annual review of phytopathology. 2012;50:155–173. 10.1146/annurev-phyto-081211-172950 22726120

[8] Thaler JS, Humphrey PT, Whiteman NK. Evolution of jasmonate and salicylate signal crosstalk. Trends in plant science. 2012;17(5):260–270. 10.1016/j.tplants.2012.02.010 22498450

[9] Müller R, De Vos M, Sun JY, Sønderby IE, Halkier BA, Wittstock U, et al Differential effects of indole and aliphatic glucosinolates on lepidopteran herbivores. Journal of chemical ecology. 2010;36(8):905–913. 10.1007/s10886-010-9825-z 20617455

[10] Kim JH, Jander G. Myzus persicae (green peach aphid) feeding on Arabidopsis induces the formation of a deterrent indole glucosinolate. The Plant Journal. 2007;49(6):1008–1019. 17257166 10.1111/j.1365-313X.2006.03019.x

[11] de Vos M, Kriksunov KL, Jander G. Indole-3-acetonitrile production from indole glucosinolates deters oviposition by *Pieris rapae* . Plant physiology. 2008;146(3):916–926. 10.1104/pp.107.112185 18192443 PMC2259081

[12] Clay NK, Adio AM, Denoux C, Jander G, Ausubel FM. Glucosinolate metabolites required for an Arabidopsis innate immune response. Science. 2009;323(5910):95–101. 10.1126/science.1164627 19095898 PMC2630859

[13] Bednarek P, Piślewska-Bednarek M, Svatoš A, Schneider B, Doubský J, Mansurova M, et al A glucosinolate metabolism pathway in living plant cells mediates broad-spectrum antifungal defense. Science. 2009;323(5910):101–106. 10.1126/science.1163732 19095900

[14] Fan J, Crooks C, Creissen G, Hill L, Fairhurst S, Doerner P, et al Pseudomonas sax genes overcome aliphatic isothiocyanate–mediated non-host resistance in arabidopsis. Science. 2011;331(6021):1185–1188. 10.1126/science.1199707 21385714

[15] Clarke DB. Glucosinolates, structures and analysis in food. Analytical Methods. 2010;2(4):310–325.

[16] Fahey JW, Zalcmann AT, Talalay P. The chemical diversity and distribution of glucosinolates and isothiocyanates among plants. Phytochemistry. 2001;56(1):5–51. 11198818 10.1016/s0031-9422(00)00316-2

[17] Grubb CD, Abel S. Glucosinolate metabolism and its control. Trends in plant science. 2006;11(2):89–100. 16406306 10.1016/j.tplants.2005.12.006

[18] Halkier BA, Gershenzon J. Biology and biochemistry of glucosinolates. Annu Rev Plant Biol. 2006;57:303–333. 16669764 10.1146/annurev.arplant.57.032905.105228

[19] Sønderby IE, Geu-Flores F, Halkier BA. Biosynthesis of glucosinolates–gene discovery and beyond. Trends in plant science. 2010;15(5):283–290. 10.1016/j.tplants.2010.02.005 20303821

[20] Liu S, Liu Y, Yang X, Tong C, Edwards D, Parkin IA, et al The *Brassica oleracea* genome reveals the asymmetrical evolution of polyploid genomes. Nature Communications. 2014;5:3930 10.1038/ncomms4930 24852848 PMC4279128

[21] Nagaharu U. Genome analysis in Brassica with special reference to the experimental formation of *B*. *napus* and peculiar mode of fertilization. Jap J Bot. 1935;7:389–452.

[22] Chalhoub B, Denoeud F, Liu S, Parkin IA, Tang H, Wang X, et al Early allopolyploid evolution in the post-Neolithic *Brassica napus* oilseed genome. Science. 2014;345(6199):950–953. 10.1126/science.1253435 25146293

[23] Wang X, Wang H, Wang J, Sun R, Wu J, Liu S, et al The genome of the mesopolyploid crop species *Brassica rapa* . Nature genetics. 2011;43(10):1035–1039. 10.1038/ng.919 21873998

[24] Li G, Quiros CF. Genetic analysis, expression and molecular characterization of *BoGSL-ELONG*, a major gene involved in the aliphatic glucosinolate pathway of Brassica species. Genetics. 2002;162(4):1937–1943. 12524361 10.1093/genetics/162.4.1937 PMC1462373

[25] Li G, Quiros C. In planta side-chain glucosinolate modification in Arabidopsis by introduction of dioxygenase Brassica homolog BoGSL-ALK. Theoretical and Applied Genetics. 2003;106(6):1116–1121. 12671761 10.1007/s00122-002-1161-4

[26] Liu Z, Hammerlindl J, Keller W, McVetty PB, Daayf F, Quiros CF, et al *MAM* gene silencing leads to the induction of C3 and reduction of C4 and C5 side-chain aliphatic glucosinolates in *Brassica napus* . Molecular breeding. 2011;27(4):467–478.

[27] Gao M, Li G, Potter D, McCombie WR, Quiros CF. Comparative analysis of methylthioalkylmalate synthase (MAM) gene family and flanking DNA sequences in *Brassica oleracea* and *Arabidopsis thaliana* . Plant cell reports. 2006;25(6):592–598. 16432629 10.1007/s00299-005-0078-1

[28] Zang YX, Kim HU, Kim JA, Lim MH, Jin M, Lee SC, et al Genome‐wide identification of glucosinolate synthesis genes in *Brassica rapa* . FEBS journal. 2009;276(13):3559–3574. 10.1111/j.1742-4658.2009.07076.x 19456863

[29] Hirani AH, Zelmer CD, McVetty PB, Daayf F, Li G. Homoeologous *GSL-ELONG* gene replacement for manipulation of aliphatic glucosinolates in *Brassica rapa* L. by marker assisted selection. Frontiers in plant science. 2013;4:55 10.3389/fpls.2013.00055 23532458 PMC3607083

[30] Hemm MR, Ruegger MO, Chapple C. The Arabidopsis *ref2* mutant is defective in the gene encoding CYP83A1 and shows both phenylpropanoid and glucosinolate phenotypes. The Plant Cell. 2003;15(1):179–194. 12509530 10.1105/tpc.006544 PMC143490

[31] Bak S, Tax FE, Feldmann KA, Galbraith DW, Feyereisen R. CYP83B1, a cytochrome P450 at the metabolic branch point in auxin and indole glucosinolate biosynthesis in Arabidopsis. The Plant Cell. 2001;13(1):101–111. 11158532 10.1105/tpc.13.1.101 PMC102201

[32] Bak S, Feyereisen R. The involvement of two P450 enzymes, CYP83B1 and CYP83A1, in auxin homeostasis and glucosinolate biosynthesis. Plant Physiology. 2001;127(1):108–118. 11553739 10.1104/pp.127.1.108 PMC117967

[33] Naur P, Petersen BL, Mikkelsen MD, Bak S, Rasmussen H, Olsen CE, et al CYP83A1 and CYP83B1, two nonredundant cytochrome P450 enzymes metabolizing oximes in the biosynthesis of glucosinolates in Arabidopsis. Plant physiology. 2003;133(1):63–72. 12970475 10.1104/pp.102.019240 PMC196579

[34] Hansen CH, Du L, Naur P, Olsen CE, Axelsen KB, Hick AJ, et al CYP83B1 is the oxime-metabolizing enzyme in the glucosinolate pathway in Arabidopsis. Journal of Biological Chemistry. 2001;276(27):24790–24796. 11333274 10.1074/jbc.M102637200

[35] Zhu B, Wang Z, Yang J, Zhu Z, Wang H. Isolation and Expression of Glucosinolate Synthesis Genes CYP83A1 and CYP83B1 in Pak Choi (*Brassica rapa* L. ssp. *chinensis* var. *communis* (N. Tsen & SH Lee) Hanelt). International journal of molecular sciences. 2012;13(5):5832–5843. 10.3390/ijms13055832 22754334 PMC3382748

[36] Douglas Grubb C, Zipp BJ, Ludwig‐Müller J, Masuno MN, Molinski TF, Abel S. Arabidopsis glucosyltransferase UGT74B1 functions in glucosinolate biosynthesis and auxin homeostasis. The Plant Journal. 2004;40(6):893–908. 15584955 10.1111/j.1365-313X.2004.02261.x

[37] Gachon CM, Langlois-Meurinne M, Henry Y, Saindrenan P. Transcriptional co-regulation of secondary metabolism enzymes in Arabidopsis: functional and evolutionary implications. Plant molecular biology. 2005;58(2):229–45. 16027976 10.1007/s11103-005-5346-5

[38] Douglas Grubb C, Zipp BJ, Kopycki J, Schubert M, Quint M, Lim EK, et al Comparative analysis of Arabidopsis UGT74 glucosyltransferases reveals a special role of UGT74C1 in glucosinolate biosynthesis. The Plant Journal. 2014;79:92–105. 10.1111/tpj.12541 24779768

[39] Kliebenstein D, Pedersen D, Barker B, Mitchell-Olds T. Comparative analysis of quantitative trait loci controlling glucosinolates, myrosinase and insect resistance in Arabidopsis thaliana. Genetics. 2002;161(1):325–332. 12019246 10.1093/genetics/161.1.325 PMC1462090

[40] Weis C, Hildebrandt U, Hoffmann T, Hemetsberger C, Pfeilmeier S, König C, et al CYP83A1 is required for metabolic compatibility of Arabidopsis with the adapted powdery mildew fungus Erysiphe cruciferarum. New Phytologist. 2014;202:1310–1319. 10.1111/nph.12759 24602105

[41] Hansen BG, Kerwin RE, Ober JA, Lambrix VM, Mitchell-Olds T, Gershenzon J, et al A novel 2-oxoacid-dependent dioxygenase involved in the formation of the goiterogenic 2-hydroxybut-3-enyl glucosinolate and generalist insect resistance in Arabidopsis. Plant physiology. 2008;148(4):2096–2108. 10.1104/pp.108.129981 18945935 PMC2593654

[42] Beekwilder J, van Leeuwen W, van Dam NM, Bertossi M, Grandi V, Mizzi L, et al The impact of the absence of aliphatic glucosinolates on insect herbivory in Arabidopsis. PLoS One. 2008;3(4):e2068 10.1371/journal.pone.0002068 18446225 PMC2323576

[43] Kroymann J, Donnerhacke S, Schnabelrauch D, Mitchell-Olds T. Evolutionary dynamics of an Arabidopsis insect resistance quantitative trait locus. Proceedings of the National Academy of Sciences. 2003;100(suppl 2):14587–14592. 10.1073/pnas.1734046100 PMC304123 14506289

[44] Züst T, Heichinger C, Grossniklaus U, Harrington R, Kliebenstein DJ, Turnbull LA. Natural enemies drive geographic variation in plant defenses. Science. 2012;338(6103):116–119. 10.1126/science.1226397 23042895

[45] Kliebenstein DJ, Rowe HC, Denby KJ. Secondary metabolites influence Arabidopsis/*Botrytis* interactions: variation in host production and pathogen sensitivity. The Plant Journal. 2005;44(1):25–36. 16167893 10.1111/j.1365-313X.2005.02508.x

[46] Cui J, Jander G, Racki LR, Kim PD, Pierce NE, Ausubel FM. Signals Involved in Arabidopsis Resistance to*Trichoplusia ni* Caterpillars Induced by Virulent and Avirulent Strains of the Phytopathogen *Pseudomonas syringae* . Plant Physiology. 2002;129(2):551–564. 12068100 10.1104/pp.010815 PMC161673

[47] Pfalz M, Vogel H, Kroymann J. The gene controlling the indole glucosinolate modifier1 quantitative trait locus alters indole glucosinolate structures and aphid resistance in Arabidopsis. The Plant Cell. 2009;21(3):985–999. 10.1105/tpc.108.063115 19293369 PMC2671713

[48] Stotz HU, Sawada Y, Shimada Y, Hirai MY, Sasaki E, Krischke M, et al Role of camalexin, indole glucosinolates, and side chain modification of glucosinolate‐derived isothiocyanates in defense of Arabidopsis against *Sclerotinia sclerotiorum* . The Plant Journal. 2011;67(1):81–93. 10.1111/j.1365-313X.2011.04578.x 21418358

[49] Tierens KF-J, Thomma BP, Brouwer M, Schmidt J, Kistner K, Porzel A, et al Study of the role of antimicrobial glucosinolate-derived isothiocyanates in resistance of Arabidopsis to microbial pathogens. Plant Physiology. 2001;125(4):1688–1699. 11299350 10.1104/pp.125.4.1688 PMC88826

[50] Sanchez‐Vallet A, Ramos B, Bednarek P, López G, Piślewska‐Bednarek M, Schulze‐Lefert P, et al Tryptophan‐derived secondary metabolites in Arabidopsis thaliana confer non‐host resistance to necrotrophic Plectosphaerella cucumerina fungi. The Plant Journal. 2010;63(1):115–127. 10.1111/j.1365-313X.2010.04224.x 20408997

[51] Bednarek P, Piślewska‐Bednarek M, Ver Loren van Themaat E, Maddula RK, Svatoš A, Schulze‐Lefert P. Conservation and clade‐specific diversification of pathogen‐inducible tryptophan and indole glucosinolate metabolism in Arabidopsis thaliana relatives. New Phytologist. 2011;192(3):713–726. 10.1111/j.1469-8137.2011.03824.x 21793828

[52] Li Y, Kiddle G, Bennett R, Wallsgrove R. Local and systemic changes in glucosinolates in Chinese and European cultivars of oilseed rape (*Brassica napus* L.) after inoculation with *Sclerotinia sclerotiorum* (stem rot). Annals of Applied Biology. 1999;134(1):45–58.

[53] Zhao J, Meng J. Detection of loci controlling seed glucosinolate content and their association with *Sclerotinia* resistance in *Brassica napus* . Plant Breeding. 2003;122(1):19–23.

[54] Fan Z, Lei W, Sun X, Yu B, Wang Y, Yang G. The association of *Sclerotinia sclerotiorum* resistance with glucosinolates in *Brassica napus* double-low dh population. Journal of Plant Pathology. 2008;90(1):43–48.

[55] Feng J, Long Y, Shi L, Shi J, Barker G, Meng J. Characterization of metabolite quantitative trait loci and metabolic networks that control glucosinolate concentration in the seeds and leaves of *Brassica napus* . New Phytologist. 2012;193(1):96–108. 10.1111/j.1469-8137.2011.03890.x 21973035

[56] Mithen R. Leaf glucosinolate profiles and their relationship to pest and disease resistance in oilseed rape. Euphytica. 1992;63(1–2):71–83.

[57] Thompson JD, Gibson TJ, Plewniak F, Jeanmougin F, Higgins DG. The CLUSTAL_X windows interface: flexible strategies for multiple sequence alignment aided by quality analysis tools. Nucleic acids research. 1997;25(24):4876–4882. 9396791 10.1093/nar/25.24.4876 PMC147148

[58] Henikoff S, Henikoff JG. Performance evaluation of amino acid substitution matrices. Proteins: Structure, Function, and Genetics. 1993;17(1):49–61. 10.1002/prot.340170108 8234244

[59] Zhou Y, Wang H, Gilmer S, Whitwill S, Keller W, Fowke LC. Control of petal and pollen development by the plant cyclin-dependent kinase inhibitor ICK1 in transgenic Brassica plants. Planta. 2002;215(2):248–257. 12029474 10.1007/s00425-002-0752-2

[60] Murray M, Thompson WF. Rapid isolation of high molecular weight plant DNA. Nucleic acids research. 1980;8(19):4321–4326. 7433111 10.1093/nar/8.19.4321 PMC324241

[61] Li Q, Yin M, Li Y, Fan C, Yang Q, Wu J, et al Expression of *Brassica napus* TTG2, a regulator of trichome development, increases plant sensitivity to salt stress by suppressing the expression of auxin biosynthesis genes. Journal of experimental botany. 2015:erv287. 10.1093/jxb/erv287 PMC4566978 26071533

[62] Zhang Y, Li B, Huai D, Zhou Y, Kliebenstein DJ. The conserved transcription factors, MYB115 and MYB118, control expression of the newly evolved benzoyloxy glucosinolate pathway in *Arabidopsis thaliana* . Frontiers in Plant Science. 2015;6:343–362. 10.3389/fpls.2015.00343 26029237 PMC4429563

[63] R Development Core Team. R: A Language and Environment for Statistical Computing, R Project for Statistical Computing, Vienna, Austria 2014;Retrieved from http://www.r-project.org.

[64] Cheng Y, Cao L, Wang S, Li Y, Shi X, Liu H, et al Downregulation of multiple CDK inhibitor ICK/KRP genes upregulates the E2F pathway and increases cell proliferation, and organ and seed sizes in Arabidopsis. The Plant Journal. 2013;75(4):642–655. 10.1111/tpj.12228 23647236

[65] Ramakers C, Ruijter JM, Deprez RHL, Moorman AF. Assumption-free analysis of quantitative real-time polymerase chain reaction (PCR) data. Neuroscience letters. 2003;339(1):62–66. 12618301 10.1016/s0304-3940(02)01423-4

[66] Kroymann J, Textor S, Tokuhisa JG, Falk KL, Bartram S, Gershenzon J, et al A gene controlling variation in Arabidopsis glucosinolate composition is part of the methionine chain elongation pathway. Plant Physiology. 2001;127(3):1077–1088. 11706188 PMC129277

[67] Li G, Riaz A, Goyal S, Abel S, Quiros C. Inheritance of three major genes involved in the synthesis of aliphatic glucosinolates in *Brassica oleracea* . Journal of the American Society for Horticultural Science. 2001;126(4):427–431.

[68] Liu Z, Hirani AH, McVetty PB, Daayf F, Quiros CF, Li G. Reducing progoitrin and enriching glucoraphanin in *Braasica napus* seeds through silencing of the GSL-ALK gene family. Plant molecular biology. 2012;79(1–2):179–189. 10.1007/s11103-012-9905-2 22477389

[69] Mikkelsen MD, Petersen BL, Glawischnig E, Jensen AB, Andreasson E, Halkier BA. Modulation of CYP79 genes and glucosinolate profiles in Arabidopsis by defense signaling pathways. Plant Physiology. 2003;131(1):298–308. 12529537 10.1104/pp.011015 PMC166809

[70] Kliebenstein DJ, Figuth A, Mitchell-Olds T. Genetic architecture of plastic methyl jasmonate responses in *Arabidopsis thaliana* . Genetics. 2002;161(4):1685–1696. 12196411 10.1093/genetics/161.4.1685 PMC1462221

[71] Kliebenstein DJ, Kroymann J, Brown P, Figuth A, Pedersen D, Gershenzon J, et al Genetic control of natural variation in Arabidopsis glucosinolate accumulation. Plant Physiology. 2001;126(2):811–825. 11402209 10.1104/pp.126.2.811 PMC111171

[72] Brown PD, Tokuhisa JG, Reichelt M, Gershenzon J. Variation of glucosinolate accumulation among different organs and developmental stages of *Arabidopsis thaliana* . Phytochemistry. 2003;62(3):471–481. 12620360 10.1016/s0031-9422(02)00549-6

[73] Nour-Eldin HH, Andersen TG, Burow M, Madsen SR, Jørgensen ME, Olsen CE, et al NRT/PTR transpcation of glucosinolate defence compounds to seeds. Nature. 2012;488:531–534. 10.1038/nature11285 22864417

