# Supplementary material for: Overexpression of Three Glucosinolate Biosynthesis Genes in Brassica napus Identifies Enhanced Resistance to Sclerotinia sclerotiorum and Botrytis cinerea
Source: PLoS One. 2015 Oct 14;10(10):e0140491. doi: 10.1371/journal.pone.0140491 (PMC4605783; doi:10.1371/journal.pone.0140491)
Supplement: S1 Table — (DOCX) [file pone.0140491.s001.docx]

**S1 Table. List of primers used in constructs and assays**

| Primer | Seguence (5' > 3') | Underlined | Destination |
| --- | --- | --- | --- |
| ***Constructs for plant transformation*** |  |  |  |
| YYp01 | TCTAGA ATGGCTTCATCGCTTCTG | *Xba*I | *35S:BnMAM1* |
| YYp02 | GAGCTC TTACACATTCGATGAAAC | *Sac*I | *35S:BnMAM1* |
| YYp07 | TCTAGAATGGCGGAAACAACAACA | *Xba*I | *35S:BnUGT74B1* |
| YYp08 | GAGCTCTCAATGTTTCTTCCCTAA | *Sac*I | *35S:BnUGT74B1* |
| YYp19 | TCTAGAATGGAAGATATCATCATCGG | *Xba*I | *35S:BrCYP83A1* |
| YYp20 | GAGCTC TTACTTGCTCACTTTCTCC | *Sac*I | *35S:BrCYP83A1* |
| ***Primers for RT-PCR*** |  |  |  |
| ActinF2 | AGCTGGAGACGGCTAAGAG |  | RT-PCR for *BnACTIN* (AF111812) |
| ActinR2 | GTTGGAAAGTGCTGAGGGA |  | RT-PCR for *BnACTIN* (AF111812) |
| YYp01-RT | GGACACTGTAGGAATCAACA |  | RT-PCR for *BnMAM1* OE plants |
| YYp02-RT | CATAGCCGTTAGTCTCTTCA |  | RT-PCR for *BnMAM1* OE plants |
| YYp07-RT | TAACCACGAAGATGCTGATT |  | RT-PCR for *BnUGT74B1* OE plants |
| YYp08-RT | TCCCTAAACTCTCCACAAAC |  | RT-PCR for *BnUGT74B1* OE plants |
| YYp19-RT | CGGCTTATATGATGGAGTGT |  | RT-PCR for *BrCYP83A1* OE plants |
| YYp20-RT | CTTGAGATGATCCGACTTGT |  | RT-PCR for *BrCYP83A1* OE plants |
| ***Primers for qRT-PCR*** |  |  |  |
| RealACT-F | CTGGAATTGCTGACCGTATGAG |  | qRT-PCR for *BnACTIN* (AF111812) |
| RealACT-R | ATCTGTTGGAAAGTGCTGAGGG |  | qRT-PCR for *BnACTIN* (AF111812) |
| RealMAM1-F | CTTATCGCCAGAAGATGTTG |  | qRT-PCR for *BnMAM1* OE plants |
| RealMAM1-R | TCATCATCCGTGATTCTCTT |  | qRT-PCR for *BnMAM1* OE plants |
| RealUGT74B1-F | TAACCACGAAGATGCTGATT |  | qRT-PCR for *BnUGT74B1* OE plants |
| RealUGT74B1-R | GGTATCATAGGTCCGATCAA |  | qRT-PCR for *BnUGT74B1* OE plants |
| RealCYP83A1-F | TCGCCTCCAAGTTCACTATA |  | qRT-PCR for *BrCYP83A1* OE plants |
| RealCYP83A1-R | CATCACTTGAGGATACTTCATC |  | qRT-PCR for *BrCYP83A1* OE plants |
| ***Primers for probe synthesis in southern blot analysis*** | |  |  |
| NPTII-F | GTGCCCTGAATGAACTGC |  | probe synthesis of *NPTII* gene |
| NPTII-R | CAATATCACGGGTAGCCA |  | probe synthesis of *NPTII* gene |
