# Supplementary material for: Overexpression of Three Glucosinolate Biosynthesis Genes in Brassica napus Identifies Enhanced Resistance to Sclerotinia sclerotiorum and Botrytis cinerea
Source: PLoS One. 2015 Oct 14;10(10):e0140491. doi: 10.1371/journal.pone.0140491 (PMC4605783; doi:10.1371/journal.pone.0140491)
Supplement: S2 Table — (DOCX) [file pone.0140491.s002.docx]

**S2 Table. GSL contents (μmol/g) of seeds from *BnMAM1*, *BnCYP83A1* and *BnUGT74B1* overexpressing T_2_ lines.**

| GSL^a^ | Wild type |  | *BnMAM1* overexpressing lines | | | |  | *BnCYP83A1* overexpressing lines | | | |  | *BnUGT74B1* overexpressing lines | | | |
| --- | --- | --- | --- | --- | --- | --- | --- | --- | --- | --- | --- | --- | --- | --- | --- | --- |
|  |  |  | OE-M-1 | *P*^b^ | OE-M-2 | *P* |  | OE-C-1 | *P* | OE-C-2 | *P* |  | OE-U-1 | *P* | OE-U-2 | *P* |
| 2OH3B | 2.60±0.13 |  | 3.66±0.17 | <0.001 | 3.52±0.15 | <0.001 |  | 3.10±0.09 | 0.006 | 3.05±0.07 | 0.012 |  | 3.05±0.10 | 0.013 | 3.10±0.13 | 0.012 |
| 3-Butenyl | 0.72±0.06 |  | 0.85±0.06 | NS^c^ | 0.79±0.07 | NS |  | 0.84±0.06 | NS | 0.82±0.04 | NS |  | 0.82±0.05 | NS | 0.83±0.06 | NS |
| 4-Pentenyl | 0.33±0.03 |  | 0.34±0.02 | NS | 0.34±0.03 | NS |  | 0.34±0.02 | NS | 0.33±0.02 | NS |  | 0.31±0.03 | NS | 0.35±0.02 | NS |
| 5MSOP | 1.00±0.04 |  | 1.1±0.08 | NS | 1.15±0.11 | NS |  | 1.15±0.07 | NS | 1.11±0.06 | NS |  | 1.08±0.05 | NS | 1.05±0.05 | NS |
| I3M | 0.36±0.03 |  | 0.34±0.04 | NS | 0.37±0.03 | NS |  | 0.36±0.03 | NS | 0.39±0.05 | NS |  | 0.50±0.04 | 0.006 | 0.48±0.04 | 0.011 |
| 4OHI3M | 1.03±0.05 |  | 1.09±0.05 | NS | 0.96±0.04 | NS |  | 1.00±0.05 | NS | 1.06±0.07 | NS |  | 1.12±0.06 | NS | 1.12±0.04 | NS |
| Total Aliphatic | 4.66±0.20 |  | 5.95±0.19 | <0.001 | 5.80±0.20 | 0.001 |  | 5.43±0.18 | 0.009 | 5.31±0.13 | 0.019 |  | 5.26±0.17 | 0.031 | 5.33±0.20 | 0.025 |
| Total Indolic | 1.39±0.05 |  | 1.43±0.07 | NS | 1.33±0.06 | NS |  | 1.36±0.07 | NS | 1.45±0.08 | NS |  | 1.61±0.07 | 0.014 | 1.61±0.07 | 0.016 |
| Total | 6.05±0.17 |  | 7.38±0.17 | <0.001 | 7.14±0.21 | 0.001 |  | 6.79±0.15 | 0.004 | 6.76±0.12 | 0.004 |  | 6.87±0.18 | 0.003 | 6.93±0.20 | 0.002 |

The data (means ±SE and *P* value, n = 18) were collected from three independent experiments and were analyzed via ANOVA.

^a^For GSL abbreviations, see Table 1.

^b^*P* value for GSL differences between the overexpression line and WT as determined by ANOVA.

^c^Not a significant *P* value (*P* > 0.05).
