# Supplementary figures and images for: Overexpression of Three Glucosinolate Biosynthesis Genes in Brassica napus Identifies Enhanced Resistance to Sclerotinia sclerotiorum and Botrytis cinerea
Source: PLoS One. 2015 Oct 14;10(10):e0140491. doi: 10.1371/journal.pone.0140491 (PMC4605783; doi:10.1371/journal.pone.0140491)

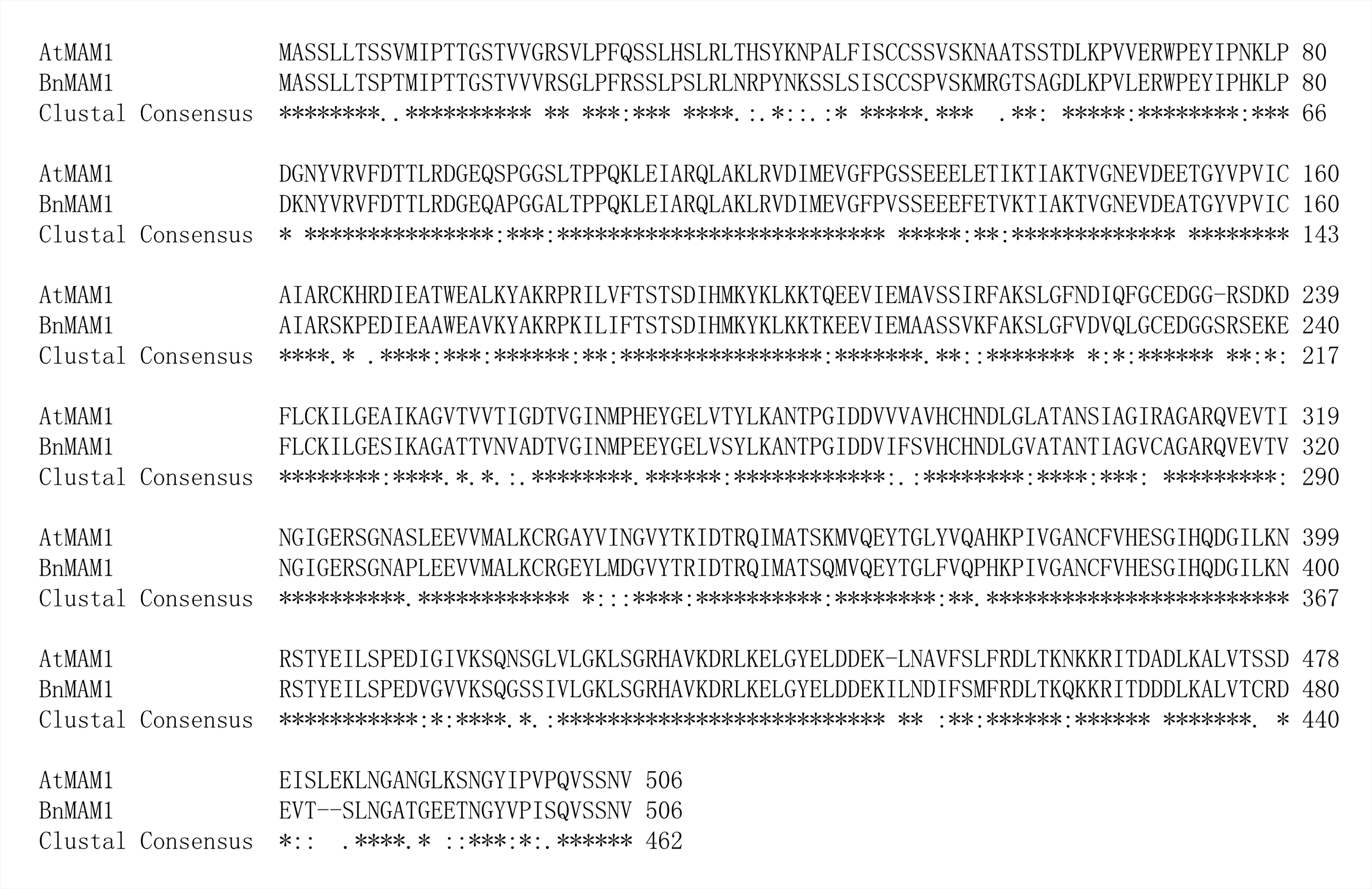

Supplement: S1 Fig — (TIF) [file pone.0140491.s003.tif]

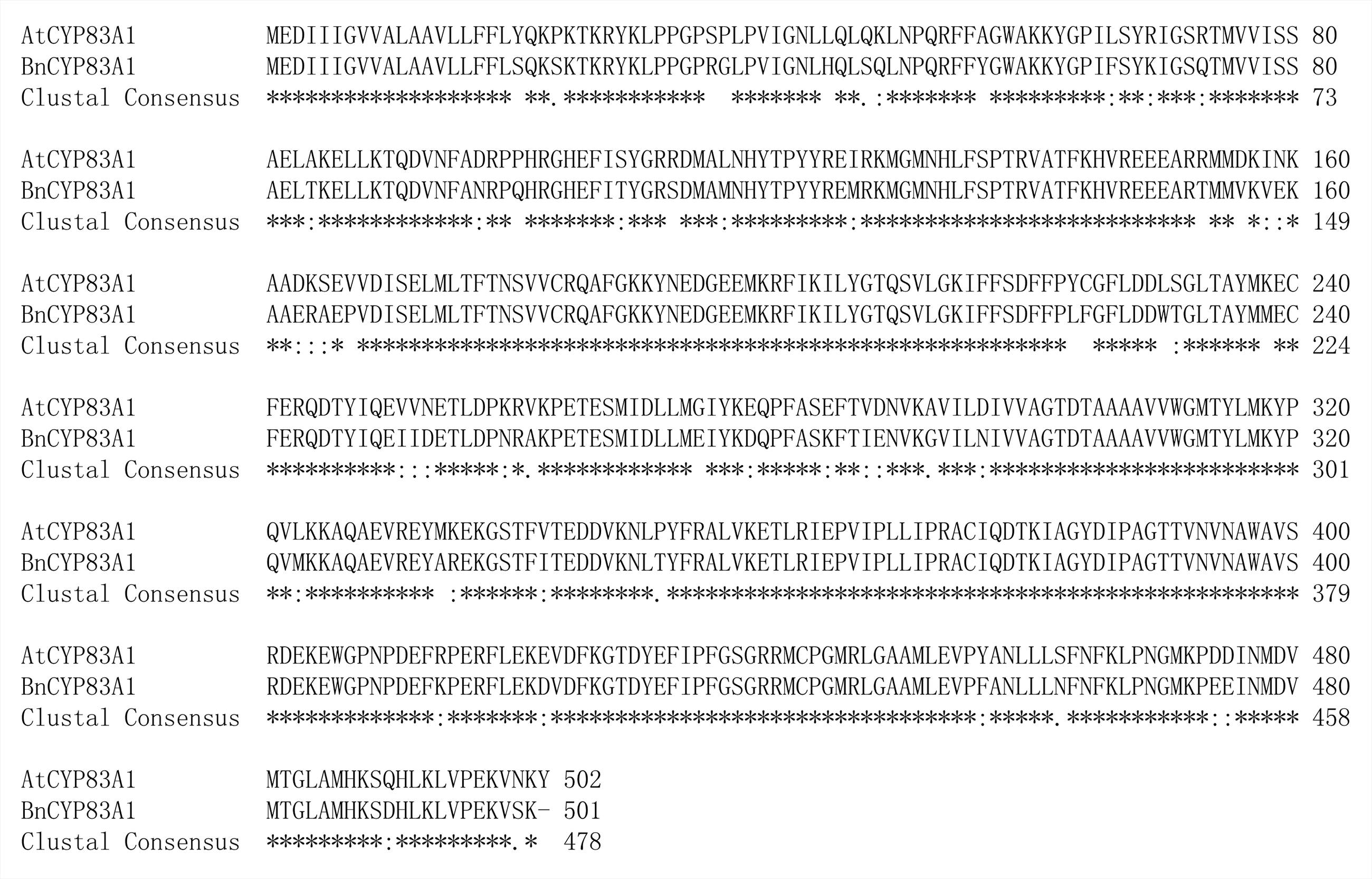

Supplement: S2 Fig — (TIF) [file pone.0140491.s004.tif]

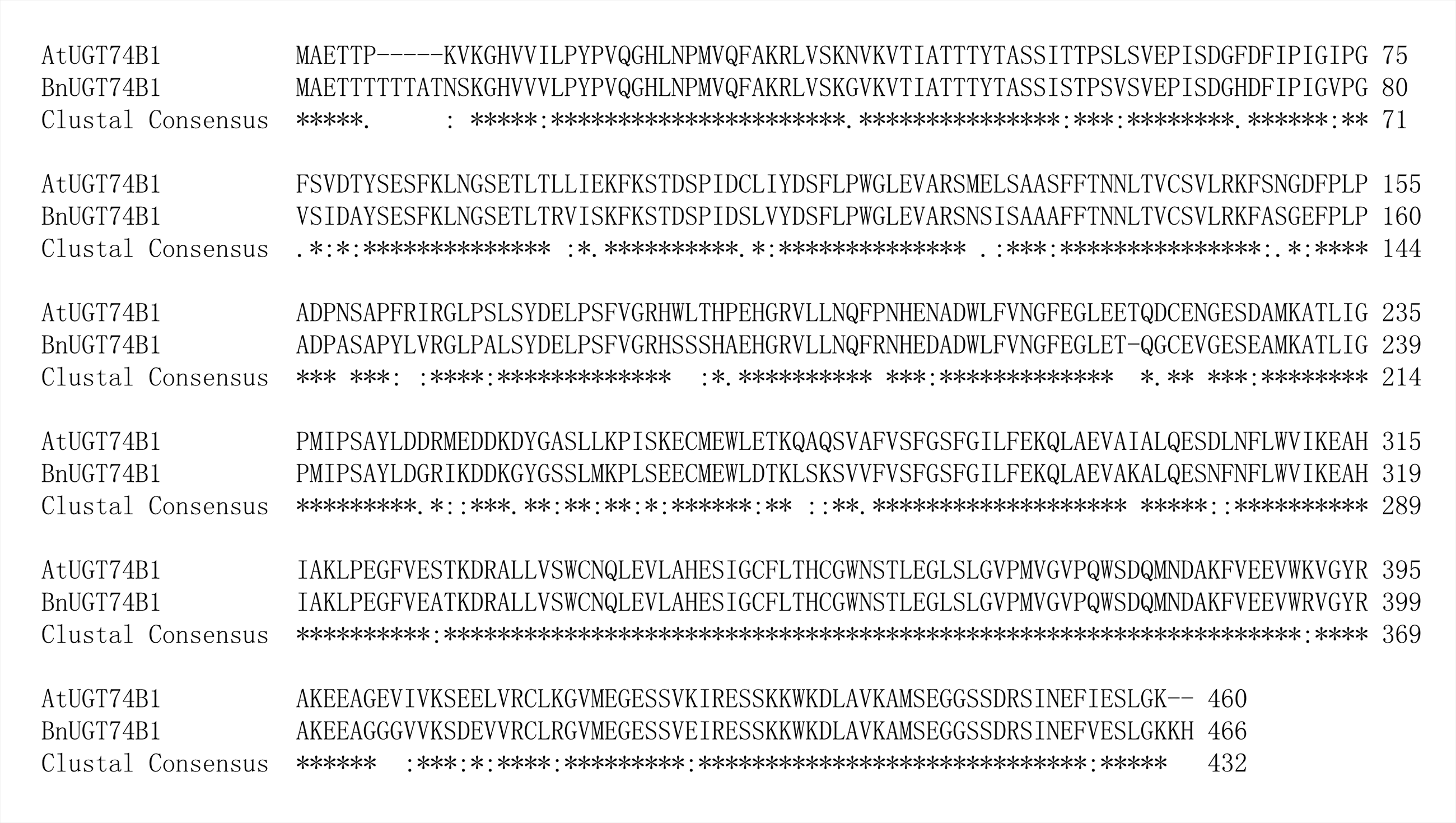

Supplement: S3 Fig — (TIF) [file pone.0140491.s005.tif]

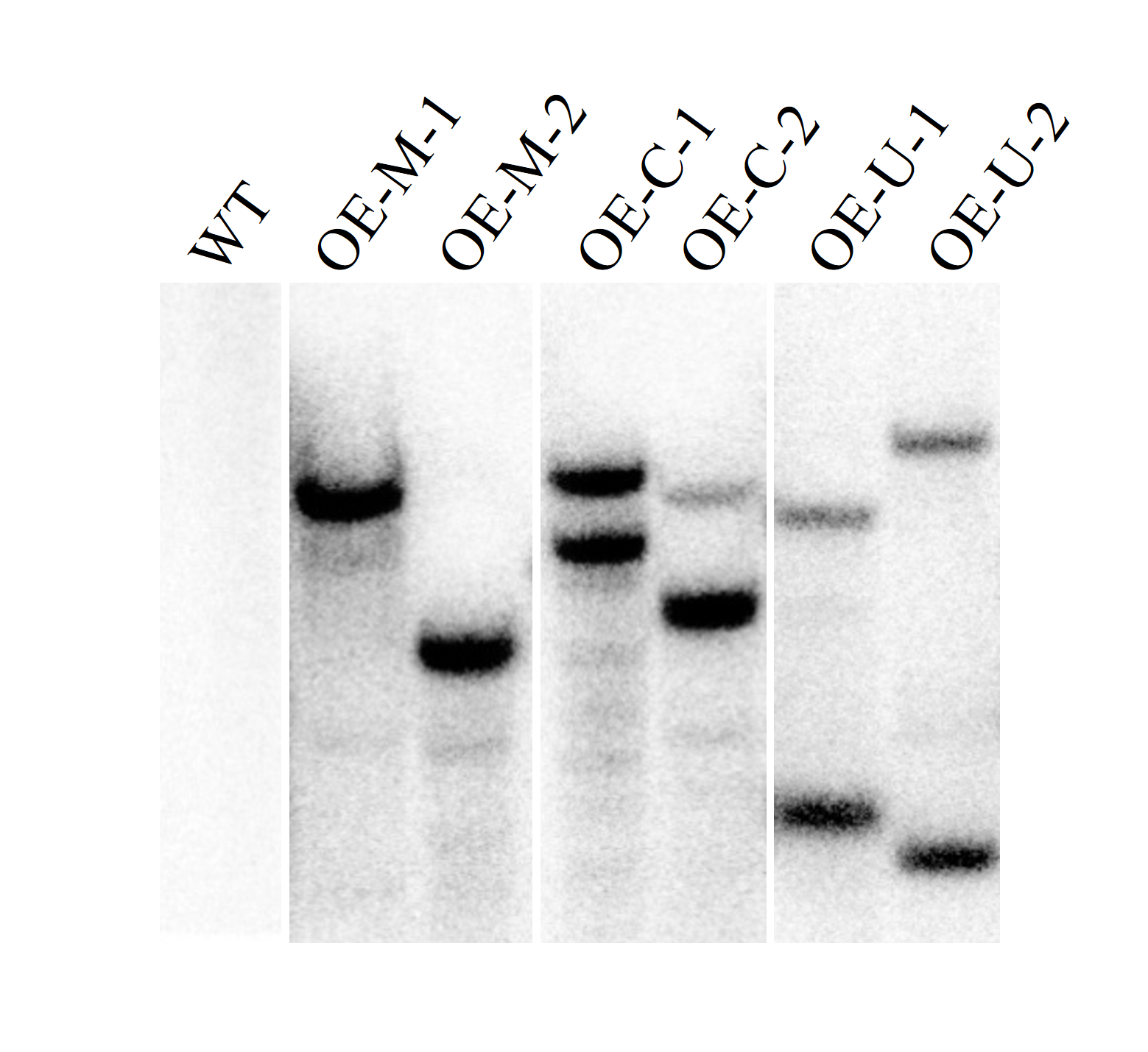

Supplement: S4 Fig — The copy number of each transgene was estimated based on the number of the bands seen on Southern blots. The genomic DNA was digested with EcoRI and a conserved 522 bp 32P-labeled NPTII 3’-terminal sequence was used as a probe. WT, untransformed wild-type control. OE- M-1 and OE-M-2 are transgenic lines for BnMAM1, OE- C-1 and OE-C-2 are transgenic lines for BnCYP83A1, OE- U-1 and OE-U-2 are transgenic lines for BnUGT74B1. (TIF) [file pone.0140491.s006.tif]
